# Reflections on Methodological Congruence in Systems and Complexity-Informed Research Comment on "What Can Policy-Makers Get Out of Systems Thinking? Policy Partners’ Experiences of a Systems-Focused Research Collaboration in Preventive Health"

**DOI:** 10.34172/ijhpm.2020.231

**Published:** 2020-11-29

**Authors:** Ana Teixeira de Melo

**Affiliations:** Centre for Social Studies, University of Coimbra, Coimbra, Portugal.

**Keywords:** Systems Thinking, Complexity Thinking, Methodological Congruence, Methodologies

## Abstract

In this paper we argue, for an increased congruence between the conceptual frameworks and the research methodology in studies focused on the theory or practice of systems and complexity-informed thinking (SCT). In doing so, we believe we can build more complex forms of knowledge with clearer and more impactful implications for practice. There is scope for both methodological innovations and the adaptation of traditional research methods to enact properties congruent with the systemic complexity of our targeted realities. We organise our reflection around the paper of Haynes et al. We provide examples of how a research methodology more deeply embedded in systems and complexity-thinking may add depth and meaning to the research results and their interpretation. We explore the creative adaptation of the interview techniques to integrate systemic forms of questioning (eg, circular and reflexive questioning) to map the patterns of interaction contributing to the outcomes of interventions.

## Introduction

In the paper titled ‘What can policy-makers get out of systems thinking? Policy Partners’ Experiences of a Systems-Focused Research Collaboration in Preventive Health,’ Haynes et al^1^ report the results of a qualitative study exploring the perceptions of policy-makers about their engagement with a systems-focused research collaboration in the domain of health.

This study offers a valuable contribution to understanding how best to engage policy-makers with systems-informed thinking. We adopt the paper of Haynes et al as a case study to explore the added value of methodological congruence in systems and complexity informed research, namely evaluation studies.^2,3^ Two preliminary notes are warranted. The first is a recognition of the value of Haynes and colleagues’ paper as it stands, and that there might have been a variety of factors and constraints at play in the definition of their methodological choices. Nevertheless, their paper offers a good opportunity to reflect about how methodological congruence in systems and complexity-informed research may contribute to building more complex knowledge (differentiated and integrated). The second point pertains to our commentary encompassing both systems and complexity thinking. Although Haynes et al paper focuses explicitly on systems thinking (ST) there are many implicit allusions to complexity, namely in their references and in terms of the approaches covered by the centre. Albeit there are significant differences between these fields, there are also relevant overlaps and we believe our reflection is equally applicable to both domains.

The main argument in this commentary is that our modes of thinking need to be congruent with the systemic complexity of the world and, hence, organised from similar principles.^2,4-6^ Such congruence may result in the emergence of information more likely to build more complex forms of knowledge (differentiated, integrated, emergent) and to provide effective guidance for action, in managing real-world complexity.^5,6^ This includes problems such as those emerging in the domain of health as well as interventions such as that mentioned in Haynes et al paper. That congruence applies to the logic underlying our methodologies.

It may be enhanced by systems and complexity thinking, namely when they result in a process of thinking that not only attends to but also enacts the same principles organising the world we aim to affect.^4,6,7^

This distinction between the level of the content (‘what’ we think about) and of the process (‘how’ we think) of the thinking is not trivial^8^ and is reflected on our methods. Haynes et al^1^ recognise this point when commenting on the results of the intervention to which their study relates.

## Methodological Congruence in Systems and Complexity-Informed Research

The study of Haynes et al,^1^ rest on the assumption that understanding and managing real-world problems such as those pertaining to health, call for a ST approach. This appears to be at the core of the Prevention Centre’s activities as a Systems-Informed Collaboration. It is not explicitly affirmed that the interventions and studies developed in the Centre integrate both the content and processes of ST, but it is likely to be so, particularly in activities with an emphasis on Soft-Systems approaches.^9^ One may assume that the interventions engaging policy-makers had a concern with not only exposing them to ST but also engaging them in such practices. This is evident in the results pertaining to changes in the modes of thinking. One may assume that, at some level, the interventions were organised according to principles compatible with ST. However, it is not clear to what extent the interventions aimed at capacity building were guided by a deep engagement with systems and complexity-informed thinking (SCT) throughout their development and implementation. It is also not clear to what extent there was an attempt to conduct a systemic conceptualisation of the processes of change — which may be associated with the desired changes — at the level of the policy-makers engagement with ST during the interventions. The authors do not report any process of eliciting/mapping factors and processes that could promote and support positive change. Knowledge of these would inform the selection and adaptation of strategies to characteristics of the participants, their work settings and contexts.

In line with the notion of methodological and theoretical congruence, one could expect that the evaluation of the Centre’s activities would rely on the recursive application of its theoretical lenses to its own operations, mapping the processes of change associated with its activities.^10^ While this might have been done, the paper makes no explicit reference to it.

At another level, the design of the study per se is not suggestive that it is embedded in a wider process that practices or applies systems or complexity-informed thinking. Our main argument is that when planning a research study, namely an intervention/evaluation study, it is necessary to consider the extent to which its methodology is embedded in a similar logic to that of the conceptual frameworks being applied to the target systems, in this case, the extent to which they enact principles from SCT. There are examples of research and evaluation proposals where this appears as a core concern.^2,10,11^

We note that the authors provided no explicit justification for targeting their research system-of-interest on the policy-makers. This kind of reflection is fundamental in a more deeply SCT research process since it has important consequences in terms of the type of understanding that may be constructed through the research process.^9,12^

Haynes et al chose a semi-structured interview of a sample of policy-makers to explore four key questions: their motivations to be involved in a systems-informed collaboration in preventive health, their experiences of ST, the factors that sustain their engagement with it, and the value they found in a ST approach.

Their approach is exploratory since there is no information about any previous process of conceptualisation that established hypotheses regarding the factors and processes that may support (more or less) positive engagement of the policy-makers. As a consequence, although the study provides interesting and useful insights which may guide future interventions, the interpretation of the results is limited in its capacity to build a broader and integrated understanding of the processes and conditions supporting positive change in relation to the participating policy-makers engagement with ST.

There are many interesting results regarding both the cases where the policy-makers expressed enthusiasm for their collaboration with the Centre and also the cases where there were more negative remarks or positions of agnosticism. However, in order to see clearer implications for future practice from these results, it would have been interesting if the paper had elaborated some hypotheses attending to then systemic patterns of interaction and the dynamics of change involved in the situation at hand.

Even when choosing to focus on individual policy-makers, it is important to have an understanding of the wider policy-making system, including its different types of actors, policies and interventions, and their internal relations, as well as the external relations to the real-world system they aim to affect. Additionally, in order to understand the possibilities for change that the intervention could have afforded, it would be necessary to consider the Centre’s activities and those of its actors, and the nature of the relations they establish with the policy-makers. It could also have been relevant to understand the wider environment in which both the policy-makers and the Centre develop their activities and the nature of the environmental constraints to which both these systems are subject to and the nature of their couplings.^5^

The questioning could then explicitly attempt to map the nature of the relations shaping the experiences of the policy-makers an even if this mapping was constructed solely from the perspective of the policy-makers themselves. Both the configurations of factors and processes that supported positive changes in the ‘enthusiastic’ as well as in the ‘agnostic’ groups of policy-makers could have been better understood through a deliberate attempt to map the conditions inhibiting and potentiating their engagement and change throughout the intervention. A more guided questioning and targeted thematic analysis could have allowed for the elaboration of hypotheses and their testing during the interview which would have afforded a better contextualisation and interpretation of the results.

This study opens opportunities to explore methodological innovations. Circular questioning is a classic assessment and intervention technique in systemic family therapy which allows for the elaboration and testing of hypotheses during therapy sessions.^13,14^

As an assessment tool, circular questioning is a way of mapping and revealing the patterns of interaction between family members that either sustain problems or may lead to their dissolution and generate new information through drawing connections and distinctions.^14^ As an intervention tool, it promotes the clients’ reflexivity in relation to its own patterns and produces information which, specific to that system, constitutes a “difference that makes a difference,” thereby freeing it to explore alternative patterns and opportunities for change. Reflexive questioning is a technique that promotes reflexivity and supports the exploration of patterns of interaction that sustain particular types of meaning and or allow for new ones.^15^

The logic associated with this kind of therapeutic approach could be of use for the SCT researcher. In the case of Haynes et al, the use of circular and reflexive questions, supported by a focus on the coupling patterns between the policy-makers and the policy-making system, the Centre (its agents, interventions, processes and activities), and their environments, could have deepened the interviewees’ reflections Interviews could have been deliberately shaped to attend to critical features of the complex organisation of the target situation producing additional about the coupling processes connecting the intervention, the policy-makers and policy-making system, their proximal contexts and the wider policies and interventions in the health domain — while themselves enacting some important properties of complex dynamical systems and supporting abductive leaps in the form of new insights.^6^

Hence, an SCT framework can guide not only the design but the *process* of conducting the study, by shaping and adapting the techniques. A traditional qualitative data collection technique can then be adapted to serve as a systemic research tool for mapping the relational organisation of the target situation and its change processes.

Box 1 and Box 2 present a list of possible circular and reflexive questions that could have guided the interviews. Questions in Box 1 focus the coupling policy-makers-intervention-contexts, and their individual contributions. Questions in Box 2 explore more dynamic aspects of change and how different configurations of conditions could be associated with different results.

**Box 1. Questions Focused on the Coupling Policy-Makers–Intervention-Context(s) and Their Respective Contributions**

- What factors and processes could have led policy-makers* not* to engage with the collaboration?
- How do policy-makers perceive how other stakeholders or relevant elements from their environments perceive and value their participation in the collaboration?
- What has contributed to the decision of those policy-makers with more negative impressions to have persisted in the collaboration?
- What could have contributed to a pre-existing interest (in the collaboration) of policy-makers?
- What kind of interventions at the level of their proximal and distant environments could have created more interest?
- What kind of arguments would have created a positive motivation for policy-makers’ engagement and for them to find fitness in the Centre’s proposals?
- In what ways would policy-makers have benefit from previous exposure to ST ideas? Which types of exposure would have been more effective and more appealing? How to reach others?
- Which aspects of existing policy-making practices are more amenable to the introduction of ST? Which are more difficult?
- In retrospect, what would have helped policy-makers make it through the ‘painful’ stage of initial engagement in a more positive way? What made them not give up at that stage?
- What would have made the development of a common language easier? How could each stakeholder have made better contributions?
- What, the behaviour of other stakeholders, made the policy-makers perceive that there was dismissal and lack of interest in non-explicitly ST ideas? What in the policy-makers response could have eased this perceived resistance?
- In what other ways could the cohesiveness and communication between stakeholders have been built during the initial stages of engagement that could have facilitated the initial “painful discussions?”
- What, in the Centre’s approach and communication strategy, could have created a greater openness and receptivity in the policy-makers? What in the policy-makers response could have made communication more positive or supported the Centre’s adaptation?
- What kind of evidence would most help policy-makers perceive the utility in ST and its tools? What kind of information is less relevant?
- What aspects of the cross-sector, co-creation process were perceived as most valuable?
- What do the policy-makers perceive as the Centre’s most significant contributions to co-creation? How were their own most significant contributions to this? What other contributions (internal or external) were relevant?
- To what extent could policy-makers have addressed the Centre in a way that would invite it to focus more on their needs? In what ways could they have presented better their positions?
- What could have allowed policy-makers to receive better and more tailored support from the Centre?

Abbreviation: ST, system thinking.

**Box 2. Questions Focused on Dynamics of Processes of Change and Configurations of Conditions and Processes**

- How have the experiences varied through time? How much have they been dependent on external/environmental circumstances?
- What would have been different in the policy-makers reports during different stages of their engagement?
- Which type of activities were more useful for whom? What role do policy-makers and the Centre have in tailoring the activities to needs? How could the collaboration be improved at that level?
- Would it have been beneficial for those policy-makers with less connection to practice to be involved in other’s experiences and projects?
- Who/which groups in the policy-makers working contexts may perceive more and less relevance in their engagement with the Centre? Who benefits the most or least? How can the benefits of ST be communicated to such audiences? What would be the relevant arguments and to what extent can the collaboration with the Centre provide them?
- What were the more significant stages for the development of shared frameworks?
- Who/which entities would have been more or less surprised with their experiences and perspectives? Who would have been more and less capable of understanding them?

The exercise renders visible e need to explore how the ‘pieces’ of information that were produced during the interview fit with each other. It would have been relevant to explore which patterns of positive and negative factors and relations could have been identified and to what extent these configurations point to prototypes that could be used to guide the adjustment and adaptation of the interventions to different types of conditions and the dynamics of the change processes.

The use of reflexive and circular questioning as investigative tools could have increased the depth of the interviews, and e interviewees’ reflexivity.

## Conclusion

In this commentary, we adopt Haynes et al paper as a case study to support our proposal that when adopting systems and complexity lenses then the thinking underlying our methodologies and methods should be embedded in the same type of logic, enacting the same type of properties. We suggest that our traditional methods (eg, data collection and analyses) can be re-invented and shaped to support more complex (differentiated, integrated, emergent) forms of knowledge, expanding our possibilities for action. Our research tools must allow us to sustain a focus on (coupling) relations as the fundamental concept to understanding the organisation of the world. In this sense, we echo Morin’s statement that “a new knowledge of organisation is capable of creating a new organisation of knowledge” (p. 358).^13^

## Acknowledgements

The author thanks Leo Caves for his preliminary feedback on the paper and for copyediting the manuscript.

## Ethical issues

Not applicable.

## Competing interests

Author declares that she has no competing interests.

## Author’s contribution

ATdM is the single author of the paper.

## Funding

The author was funded by the Portuguese Foundation for Science and Technology, under the Transition Norm (DL/57/2016/CP1341/CT0011).
